# Supplementary material for: Engineering and evaluation of FXa bypassing agents that restore hemostasis following Apixaban associated bleeding
Source: Nat Commun. 2024 May 9;15:3912. doi: 10.1038/s41467-024-48278-1 (PMC11082157; doi:10.1038/s41467-024-48278-1)
Supplement: Supplementary file 1 — Supplementary Information [file 41467_2024_48278_MOESM1_ESM.pdf]

Supplementary Information

Engineering and evaluation of FXa bypassing agents that restore hemostasis following Apixaban associated bleeding.

Wojciech Jankowski[1, #], Stepan S. Surov[1, #], Nancy E. Hernandez[1], Atul Rawal[1], Marcos Battistel[2], Daron Freedberg[2], Mikhail V. Ovanesov[1], Zuben E. Sauna[1, *]

[1] Hemostasis Branch 1, Division of Hemostasis, Office of Plasma Protein Therapeutics, Office of Therapeutic Products, Center for Biologics Evaluation & Research, US FDA, Silver Spring, MD, 20993 USA, [2] Laboratory of Bacterial Polysaccharides, Division of Bacterial, Parasitic and Allergenic Products, Office of Vaccines Research and Review, Center for Biologics Evaluation & Research, US FDA, Silver Spring, MD, 20993 USA

[#]Equal contribution
[*]Corresponding author
email: Zuben.Sauna@fda.hhs.gov

Variant	Sequence
RDR2_1	ANSFLEEMKKGHLERECMEETCSYEEAREVFEDSDKTNEFWNKYKDGDQCETSPCQNQGKCKDGLGEYTCTCLEGFEGKNCELFTRKLCSLDNGDCDQFCHEEQNSVVCSCAR<br>GYTLADNGKACIPTGPYPCGKQTLERRKRSVAQATSSSGEAPDSITWKPYDAADLDPTENPFDLLDFNQTQPERGDNNLTRIVGGQECKDGECPWQALLINEENEGFCGGTIL<br>SEFYILTAAHCLYQAKRFKVRVGDRNTEQEEGGEAVHEVEVVIKHNRFTKETYDFDIAVLRLKTPITFRMNVAPACLPERDWAESTLMTQKTGIVSGFGRTHEKGRQSTRLKM<br>LEVPYVDRNSCKLSSS**W**IITQNMFCAGYDTKQEDACQGDSGGPHVTRFKDTYFVTGIVSWGEGCARKGKYGIYTKVTAFLKWIDRSMKTRGLPKAKSHAPEVITSSPLK
RDR2_2	ANSFLEEMKKGHLERECMEETCSYEEAREVFEDSDKTNEFWNKYKDGDQCETSPCQNQGKCKDGLGEYTCTCLEGFEGKNCELFTRKLCSLDNGDCDQFCHEEQNSVVCSCAR<br>GYTLADNGKACIPTGPYPCGKQTLERRKRSVAQATSSSGEAPDSITWKPYDAADLDPTENPFDLLDFNQTQPERGDNNLTRIVGGQECKDGECPWQALLINEENEGFCGGTIL<br>SEFYILTAAHCLYQAKRFKVRVGDRNTEQEEGGEAVHEVEVVIKHNRFTKETYDFDIAVLRLKTPITFRMNVAPACLPERDWAESTLMTQKTGIVSGFGRTHEKGRQSTRLKM<br>LEVPYVDRNSCKLSSSFIITQNMFCAGYDTKQEDACQGDSGGPHVTRFKDTYFVTGIVS**A**GEGCARKGKYGIYTKVTAFLKWIDRSMKTRGLPKAKSHAPEVITSSPLK
RDR2_3	ANSFLEEMKKGHLERECMEETCSYEEAREVFEDSDKTNEFWNKYKDGDQCETSPCQNQGKCKDGLGEYTCTCLEGFEGKNCELFTRKLCSLDNGDCDQFCHEEQNSVVCSCAR<br>GYTLADNGKACIPTGPYPCGKQTLERRKRSVAQATSSSGEAPDSITWKPYDAADLDPTENPFDLLDFNQTQPERGDNNLTRIVGGQECKDGECPWQALLINEENEGFCGGTIL<br>SEFYILTAAHCLYQAKRFKVRVGDRNTEQEEGGEAVHEVEVVIKHNRFTKETYDFDIAVLRLKTPITFRMNVAPACLPERDWAESTLMTQKTGIVSGFGRTHEKGRQSTRLKM<br>LEVPYVDRNSCKLSSSFIITQNMFCAGYDTKQEDACQGDSGGPHVTRFKDTYFVTGIVSWGEGCARKGKY**V**IYTKVTAFLKWIDRSMKTRGLPKAKSHAPEVITSSPLK
RDR2_4	ANSFLEEMKKGHLERECMEETCSYEEAREVFEDSDKTNEFWNKYKDGDQCETSPCQNQGKCKDGLGEYTCTCLEGFEGKNCELFTRKLCSLDNGDCDQFCHEEQNSVVCSCAR<br>GYTLADNGKACIPTGPYPCGKQTLERRKRSVAQATSSSGEAPDSITWKPYDAADLDPTENPFDLLDFNQTQPERGDNNLTRIVGGQECKDGECPWQALLINEENEGFCGGTIL<br>SEFYILTAAHCLYQAKRFKVRVGDRNTEQEEGGEAVHEVEVVIKHNRFTKETYDFDIAVLRLKTPITFRMNVAPACLPERDWAESTLMTQKTGIVSGFGRTHEKGRQSTRLKM<br>LEVPYVDRNSCKLSSSFIITQNMFCAGYDTKQEDACQGDSGGPHVTRFKDTYFVTGIVSWGEGCARKGKY**A**IYTKVTAFLKWIDRSMKTRGLPKAKSHAPEVITSSPLK
RDR2_5	ANSFLEEMKKGHLERECMEETCSYEEAREVFEDSDKTNEFWNKYKDGDQCETSPCQNQGKCKDGLGEYTCTCLEGFEGKNCELFTRKLCSLDNGDCDQFCHEEQNSVVCSCAR<br>GYTLADNGKACIPTGPYPCGKQTLERRKRSVAQATSSSGEAPDSITWKPYDAADLDPTENPFDLLDFNQTQPERGDNNLTRIVGGQECKDGECPWQALLINEENEGFCGGTIL<br>SEFYILTAAHCLYQAKRFKVRVGDRNTEQEEGGEAVHEVEVVIKHNRFTKETYDFDIAVLRLKTPITFRMNVAPACLPERDWAESTLMTQKTGIVSGFGRTHEKGRQSTRLKM<br>LEVPYVDRNSCKLSSSFIITQNMFCAGYDTKQEDACQGDSGGPHVTRFKDTYFVTGIVSW**V**EGCARKGKYGIYTKVTAFLKWIDRSMKTRGLPKAKSHAPEVITSSPLK
RDR2_6	ANSFLEEMKKGHLERECMEETCSYEEAREVFEDSDKTNEFWNKYKDGDQCETSPCQNQGKCKDGLGEYTCTCLEGFEGKNCELFTRKLCSLDNGDCDQFCHEEQNSVVCSCAR<br>GYTLADNGKACIPTGPYPCGKQTLERRKRSVAQATSSSGEAPDSITWKPYDAADLDPTENPFDLLDFNQTQPERGDNNLTRIVGGQECKDGECPWQALLINEENEGFCGGTIL<br>SEFYILTAAHCLYQAKRFKVRVGDRNTEQEEGGEAVHEVEVVIKHNRFTKETYDFDIAVLRLKTPITFRMNVAPACLPERDWAESTLMTQKTGIVSGFGRTHEKGRQSTRLKM<br>LEVPYVDRNSCKLSSSFIITQNMFCAGYDTKQEDACQGDSGGPHVTRFKDTYFVTGIVSW**A**EGCARKGKYGIYTKVTAFLKWIDRSMKTRGLPKAKSHAPEVITSSPLK
RDR2_7	ANSFLEEMKKGHLERECMEETCSYEEAREVFEDSDKTNEFWNKYKDGDQCETSPCQNQGKCKDGLGEYTCTCLEGFEGKNCELFTRKLCSLDNGDCDQFCHEEQNSVVCSCAR<br>GYTLADNGKACIPTGPYPCGKQTLERRKRSVAQATSSSGEAPDSITWKPYDAADLDPTENPFDLLDFNQTQPERGDNNLTRIVGGQECKDGECPWQALLINEENEGFCGGTIL<br>SEFYILTAAHCLYQAKRFKVRVGDRNTEQEEGGEAVHEVEVVIKHNRFTKETYDFDIAVLRLKTPITFRMNVAPACLPERDWAESTLMTQKTGIVSGFGRTHEKGRQSTRLKM<br>LEVPYVDRNSCKLSSSFIITQNMFCAGYDTKQEDA**A**QGDSGGPHVTRFKDTYFVTGIVSWGEGCARKGKYGIYTKVTAFLKWIDRSMKTRGLPKAKSHAPEVITSSPLK
RDR2_8	ANSFLEEMKKGHLERECMEETCSYEEAREVFEDSDKTNEFWNKYKDGDQCETSPCQNQGKCKDGLGEYTCTCLEGFEGKNCELFTRKLCSLDNGDCDQFCHEEQNSVVCSCAR<br>GYTLADNGKACIPTGPYPCGKQTLERRKRSVAQATSSSGEAPDSITWKPYDAADLDPTENPFDLLDFNQTQPERGDNNLTRIVGGQECKDGECPWQALLINEENEGFCGGTIL<br>SEFYILTAAHCLYQAKRFKVRVGDRNTEQEEGGEAVHEVEVVIKHNRFTKETYDFDIAVLRLKTPITFRMNVAPACLPERDWAESTLMTQKTGIVSGFGRTHEKGRQSTRLKM<br>LEVPYVDRNSCKLSSSFIITQNMFCAGYDTKQEDACQGDSGGPHVTRFKDTYFVTGIVSWGEG**A**ARKGKYGIYTKVTAFLKWIDRSMKTRGLPKAKSHAPEVITSSPLK
RDR2_9	ANSFLEEMKKGHLERECMEETCSYEEAREVFEDSDKTNEFWNKYKDGDQCETSPCQNQGKCKDGLGEYTCTCLEGFEGKNCELFTRKLCSLDNGDCDQFCHEEQNSVVCSCAR<br>GYTLADNGKACIPTGPYPCGKQTLERRKRSVAQATSSSGEAPDSITWKPYDAADLDPTENPFDLLDFNQTQPERGDNNLTRIVGGQECKDGECPWQALLINEENEGFCGGTIL<br>SEFYILTAAHCLYQAKRFKVRVGDRNTEQEEGGEAVHEVEVVIKHNRFTKETYDFDIAVLRLKTPITFRMNVAPACLPERDWAESTLMTQKTGIVSGFGRTHEKGRQSTRLKM<br>LEVPYVDRNSCKLSSSFIITQNMFCAGYDTKQEDA**A**QGDSGGPHVTRFKDTYFVTGIVSWGEG**A**ARKGKYGIYTKVTAFLKWIDRSMKTRGLPKAKSHAPEVITSSPLK
HI_8	ANSFLEEMKKGHLERECMEETCSYEEAREVFEDSDKTNEFWNKYKDGDQCETSPCQNQGKCKDGLGEYTCTCLEGFEGKNCELFTRKLCSLDNGDCDQFCHEEQNSVVCSCAR<br>GYTLADNGKACIPTGPYPCGKQTLERRKRSVAQATSSSGEAPDSITWKPYDAADLDPTENPFDLLDFNQTQPERGDNNLTRIVGGQECKDGECPWQALLINEENEGFCGGTIL<br>SEFYILTAAHCLYQAKRFKVRVGDRNTEQEEGGEAVHEVEVVIKH**STYVPG**TYDFDIAVLRLKTPITFRMNVAPACLPERDWAESTLMTQKTGIVSGFGRTHEKGRQSTRLKM<br>LEVPYVDRNSCKLSSSFIITQNMFCAGYDTKQEDACQGDSGGPHVTRFKDTYFVTGIVSWGEGCARKGKYGIYTKVTAFLKWIDRSMKTRGLPKAKSHAPEVITSSPLK

Supplementary Table 1. Amino acid sequences of FX variants. Amino acid changes are depicted in red bold.

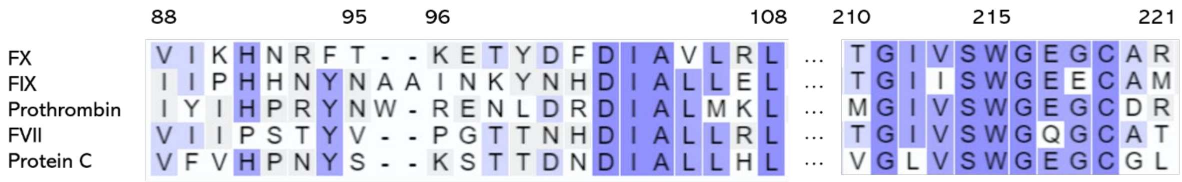

Supplementary Figure 1. An alignment of region 88-108 and 210-221 of human FX with other human vitamin K-dependent serine proteases.

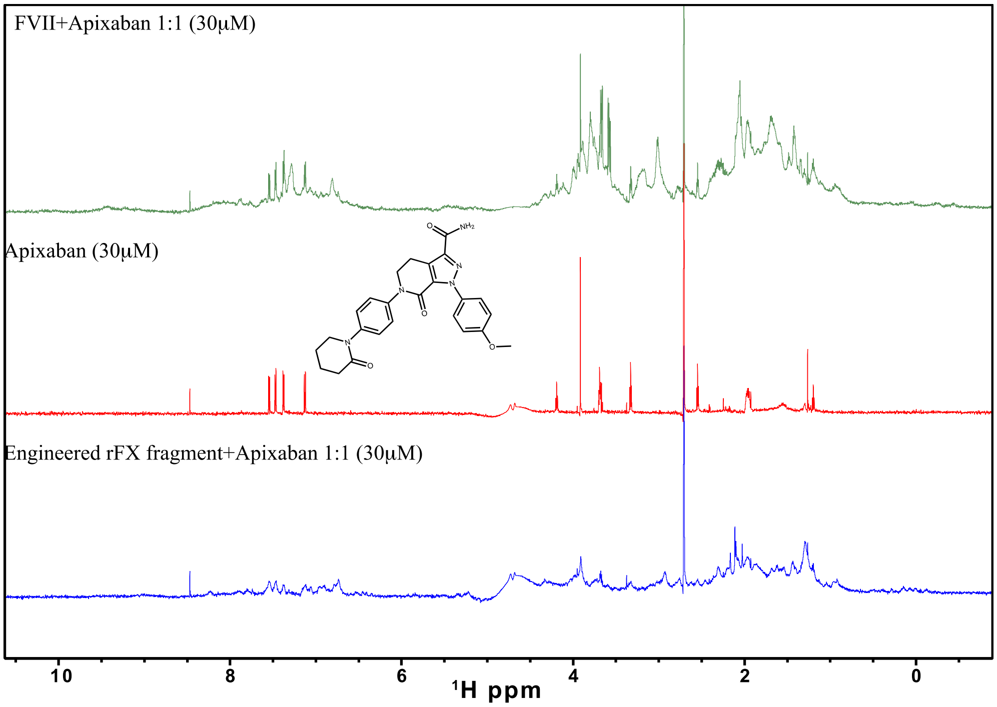

Supplementary Figure 2. ^{1}H NMR stacked spectra of Factor VIIa (FVIIa) with stoichiometric amounts of apixaban (green), negative control sample; apixaban (red), reference spectrum; and recombinant (r)FXa:apixaban in stoichiometric proportions (blue), test sample. The green spectrum is the result of superposition of ligand and protein signals with no alteration. The reference, red NMR spectrum, contains only sharp signals, as expected for an NMR spectrum of a small molecule. However, even though there is a small molecule in the sample, the blue ^{1}H NMR spectrum shows only broadened NMR signals, indicative of strong Apixaban binding to FXa. These spectra show strong binding of apixaban to FXa but no binding of apixaban to FVIIa.

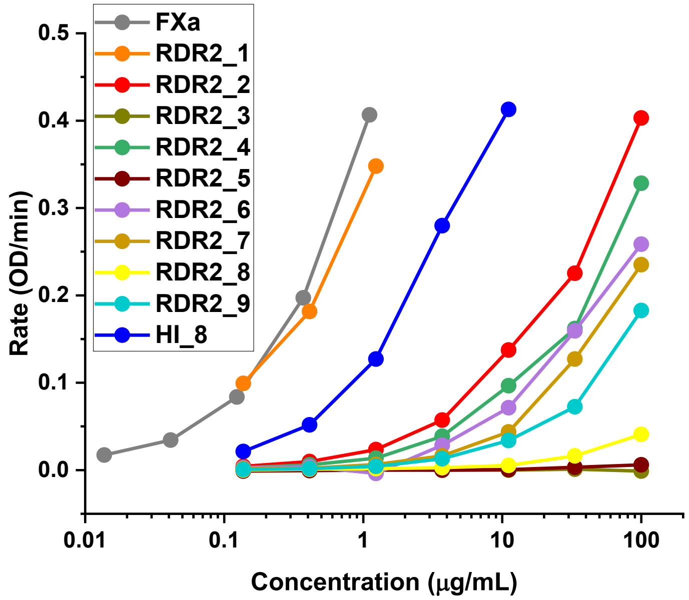

Supplementary Figure 3. The rate of chromogenic substrate cleavage in buffered purified protein system by FXa variants at different concentrations. This assay was used for screening purposes in which each variant was tested once.

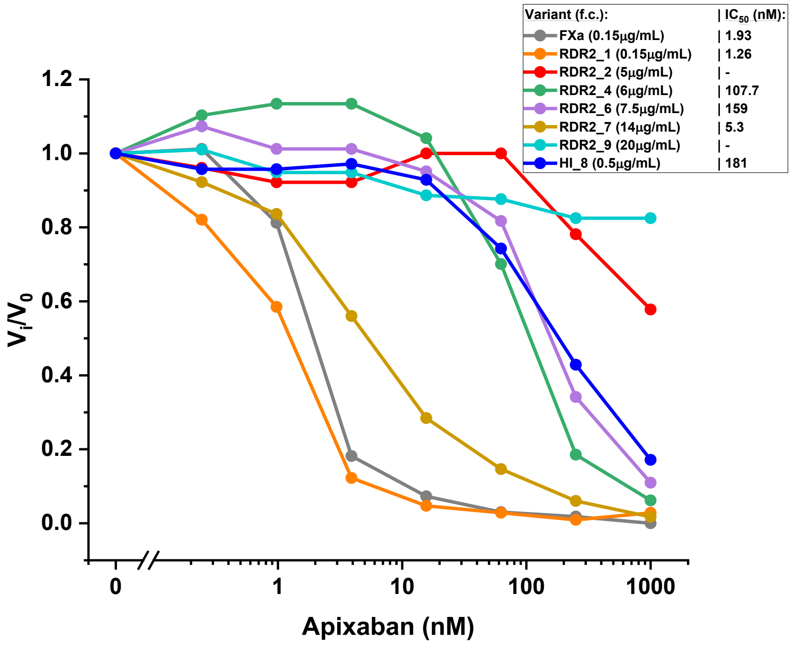

Supplementary Figure 4. The rate of chromogenic substrate cleavage by designated concentrations of FXa variants in the absence (Vo) or presence (Vi) of increasing apixaban concentrations. This assay was used for screening purposes in which each variant was tested once.

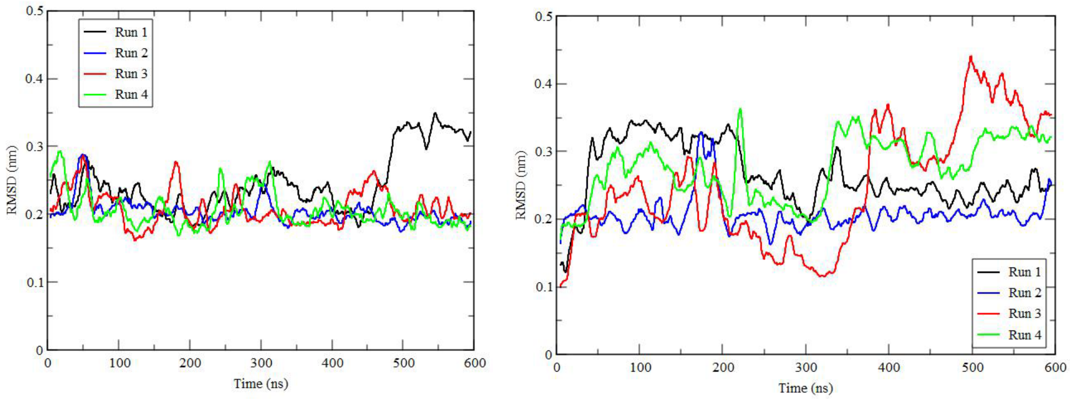

Supplementary Figure 5. Root mean square deviation for wt-FXa (left) and HI_8 (right) from four simulation runs. To investigate the molecular behavior responsible for the resistance of the apixaban for the variants, RMSD curves were generated for both the isoform wt-FXa and HI_8 homology insert region. These yeilded a displacement of the loop for all four trial runs as seen by the large deviations for the region throughout the 600ns simulations. Notable movement of the homology insert region is observed in all four simulation runs for the HI_8 structure when compared to the wt-FXa, which had a more stable RMSD throughout the 600ns simulation period. This suggests increased (compared to wt-FXa) mobility of the homology insert region of the HI_8 structure. The increased mobility could result in steric hindrance between the flexible region and apixaban (or any drug), impeding the binding between the drug and the protein.

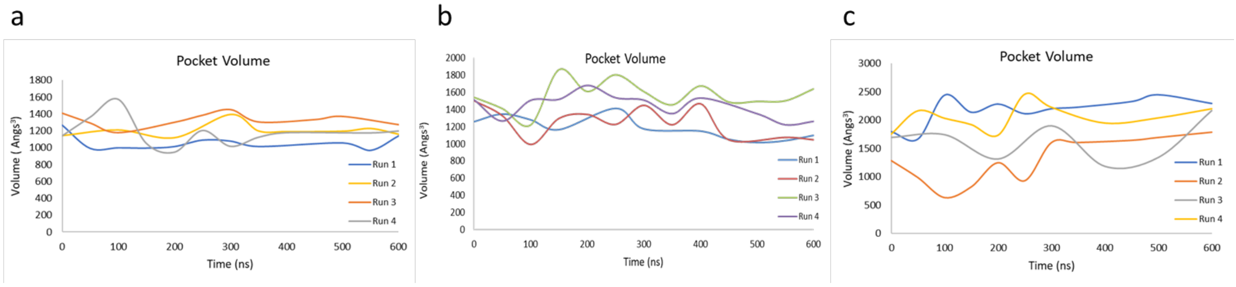

Supplementary Figure 6. Pocket volume for wt-FXa (a), HI_8 (b), and RDR2_2 (c) for the 600ns simulation period. Pocket volume calculations were performed for all three structures for the apixaban binding pocket within the protein. HI_8 and RDR2_2 had more pronounced volume deviations throughout the 600ns simulation period for all four simulation runs, compared to the wt-FXa, which had a more stable pocket volume throughout. Compared to wt-FXa, a decrease in the pocket volume is observed for HI_8, whereas an increase is observed for the RDR2_2. The notable movement of the homology insert region for HI_8 closer to the pocket could be responsible for the decrease in the pocket volume. The increase in the pocket volume for the RDR2_2, on the other hand, could be attributed to the substituion of the larger Thr with a smaller Ala residue.

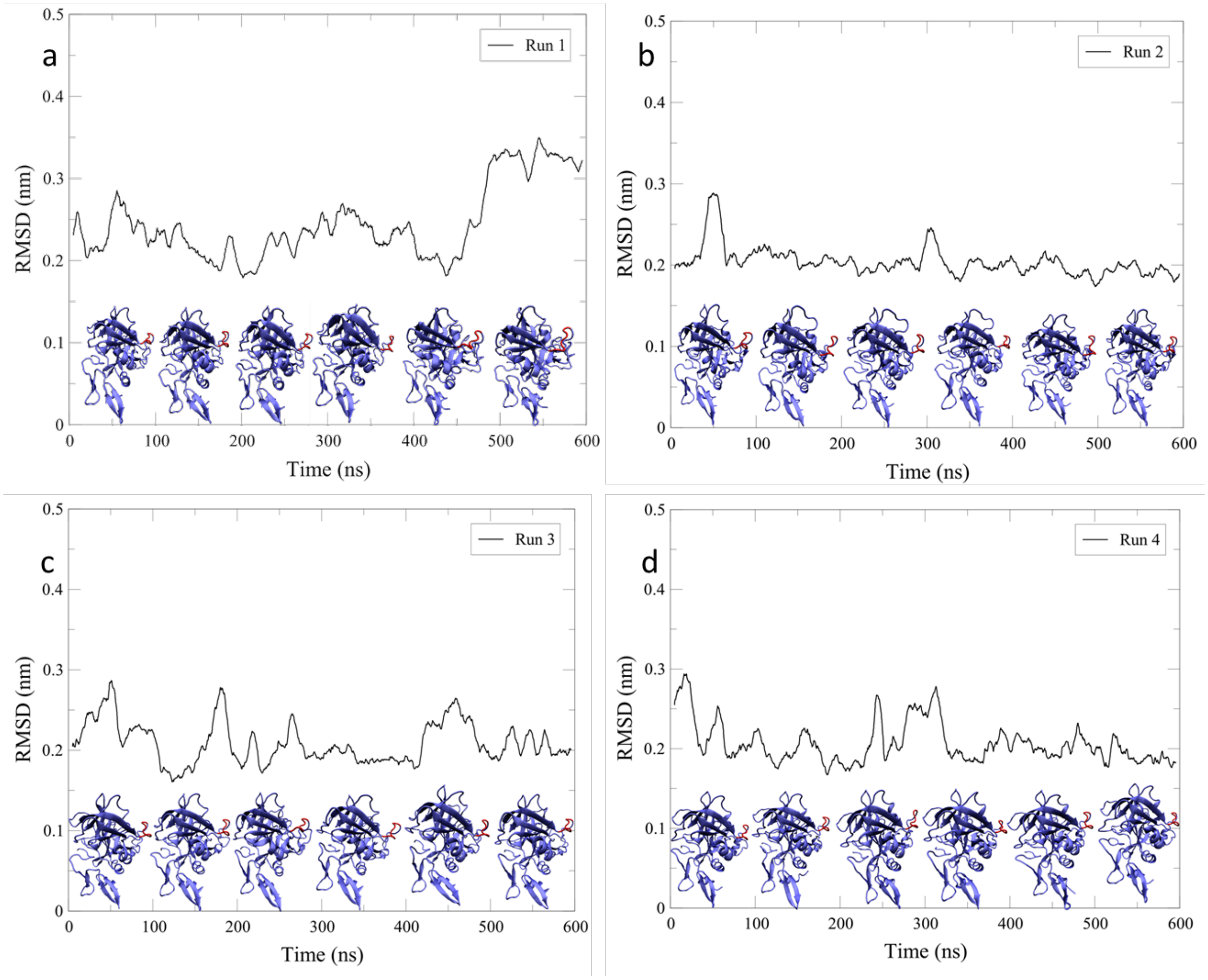

Supplementary Figure 7. Root mean square deviation along with trajectory snapshots for four wt-FXa simulation runs (a, b, c and d) taken at intervals of 0 ns, 200 ns, 300 ns, 400 ns, 500 ns and 600 ns.

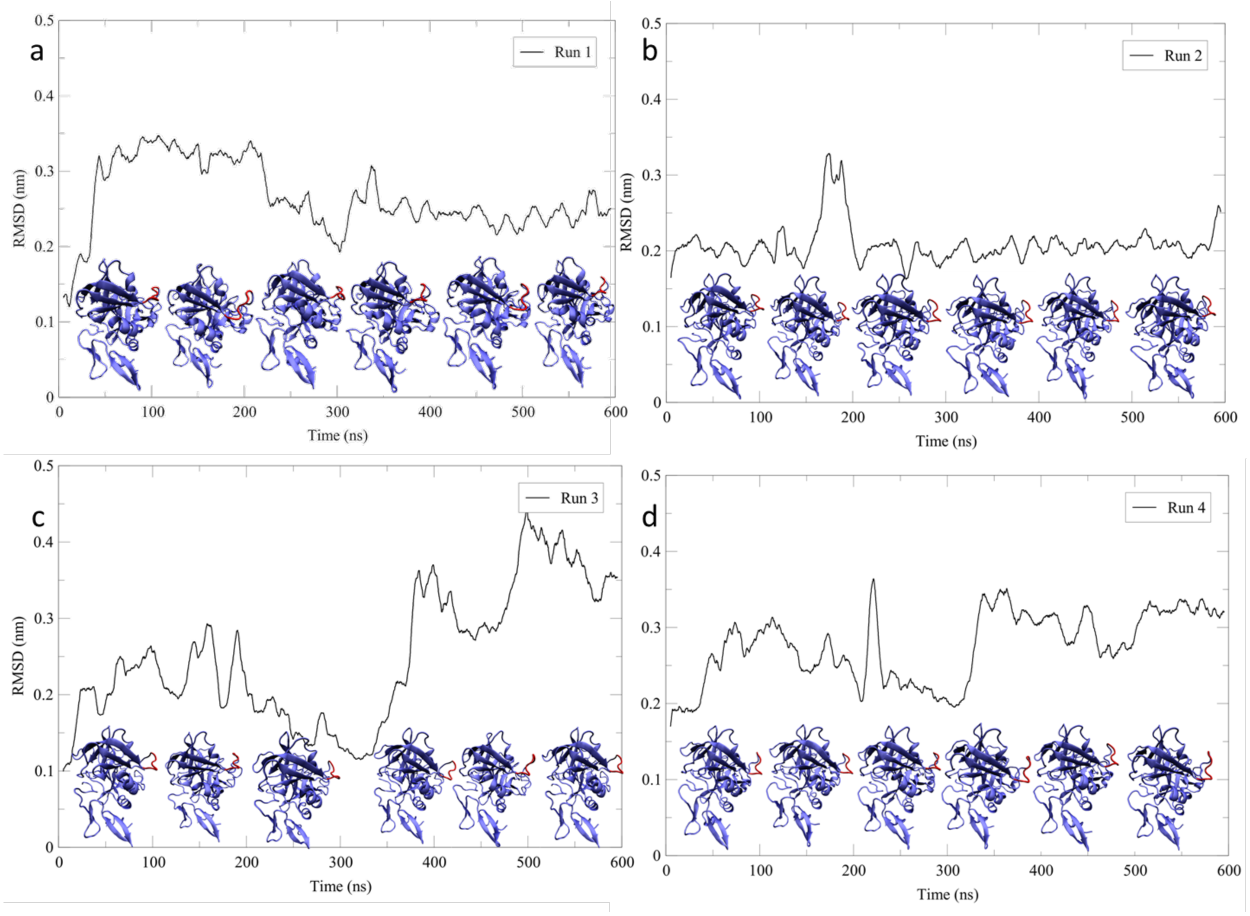

Supplementary Figure 8. Root mean square deviation along with trajectory snapshots for four HI_8 simulation runs (a, b, c and d). The flexibility of the homology insert region (red) is highlighted. Snapshots were taken at 0 ns, 200 ns, 300 ns, 400 ns, 500 ns and 600 ns.

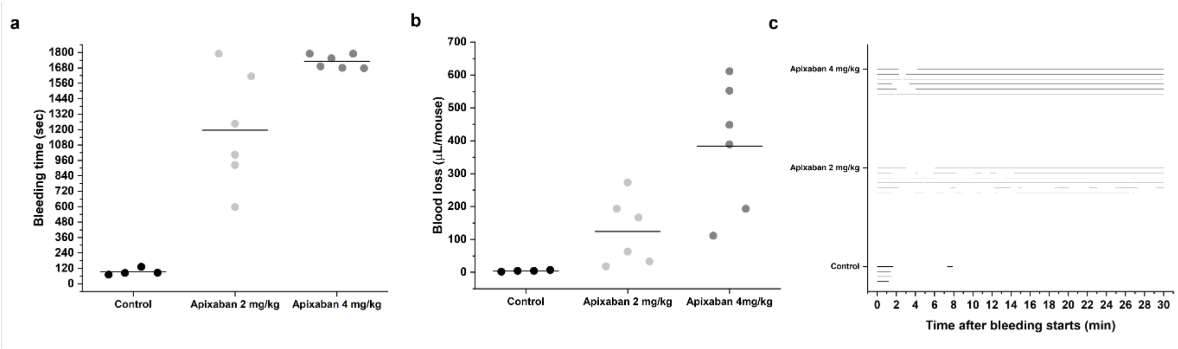

Supplementary Figure 9. Apixaban dose determination in mice tail-clipping model. Bleeding time (a), blood loss (b) and bleeding profiles (c) in mice treated with diluent (Control, n=4) or the indicated concentrations of apixaban (n=6 per group). Black horizontal lines (a, b) indicate means within each group. These data were used to determine the concentration of Apixaban to be used in the animal studies depicted in Figure 4 (main text).

Computational scripts and files

The following scripts detail the use of the RosettaRelax protocol to prepare an input structure (typically crystal structure) before use in the RosettaDesign protocol. The RosettaRelax protocol samples conformations of a given structure to find the lowest scoring variant with coordinates constrained to the starting crystal structure coordinates. This is common practice and allows for a fair comparison between structures. Typically, Rosetta allows for RosettaRelax and RosettaDesign using an XML file. The "SimpleFRFX_noligand.xml" was used for RosettaRelax (with atom constraints) while the "DesignSimpleFRFX_noligand.xml" was used for RosettaDesign (without atom constraints, to allow for more freedom and movement in the FXa protein). Both XMLs are tailored to the FXa PDB 2P16 structure. For example, the cavity is selected in "<Index name="cavity" resnums="1,2,27,42,80-86,127,131,132,134-137,139,146,148,160-165,168-172,178-189,202-211,214-219" />", which is a way to tell Rosetta these are the cavity residues and subsequentially later are used in TaskOperations, Filters, and Movers. Different XMLs were used depending on the method, RosettaRelax and RosettaDesign. The "<PROTOCOLS>" section for each XML details in order what actions Rosetta is taking. The scripts are provided so that the reader may take them directly and modify the logic to their protein of interests. The computational metrics used were total_score, change in total_score (compared to the input structure), cavity_score, 2nd-shell_score, and RMSD_WT. Total_score and cavity_score were used to determine top-scoring designs. Finally, the manual inspection of the top-scoring designs was performed by superimposing the FXa-no apixaban designs with the FXa-apixaban-threaded designs. This allowed for identification of clashes with apixaban, any new novel interactions, if mutations introduced favorable hydrophobic interactions that could potentially compete with apixaban binding by space filling, and any favorable electrostatics in the apixaban binding pocket.

Section 1: Rosetta Relax

Following are scripts and input files used in the FastRelax protocol:

Input PDB file is provided as separate file:

2p16_cp_new_noligand.pdb

Command_no_ligand.sh

```
for i in {1..50}
  do
    (bash noligand_FR.sh 2p16_cp_new_noligand.pdb 2p16_noligand.res _noligand_$i >
2p16_FR_noligand_Log.txt) &
  done
```

noligand_FR.sh

```
RosettaPath/bin/rosetta_scripts.linuxgccrelease @noligand_FR_general.flags -s $1 -out:pdb_gz true -
parser:script_vars resfile=$2 -suffix $3
```

noligand_FR.flags

```
-parser:protocol path_to_XML/SimpleFRFX_noligand.xml
-packing
 -ex1
 -ex2
#-ex2aro
#-ex1aro
 -extrachi_cutoff 0
 -use_input_sc
-run:preserve_header
-overwrite
-database path_to_rosetta_database/
-score:weights beta_nov16_cst
-in:ignore_unrecognized_res
-out::file::output_virtual
-ignore_zero_occupancy false
-restore_talaris_behavior
-out:nstruct 1
```

SimpleFRFX_noligand.xml

```
<ROSETTASCRIPTS>
  <SCOREFXNS>

    <ScoreFunction name="myscore" weights="talaris2013_cst.wts" symmetric="0">
      <Reweight scoretype="coordinate_constraint" weight="10.0" />
    </ScoreFunction>

  </SCOREFXNS>
  <RESIDUE_SELECTORS>

    <Index name="cavity" resnums="1,2,27,42,80-86,127,131,132,134-137,139,146,148,160-165,168-
172,178-189,202-211,214-219" />
    <Neighborhood name="near_cavity" selector="cavity" distance="10.0" />
   <Not name="non_cavity" selector="cavity" />
    <Or name="cavity_n_near_cavity" selectors="cavity,near_cavity" />
    <Not name="non_cavity_n_near_cavity" selector="cavity_n_near_cavity" />
    <And name="2nd_shell" selectors="non_cavity,cavity_n_near_cavity" />
    <Not name="non_2nd_shell" selector="2nd_shell" />

  </RESIDUE_SELECTORS>

  <TASKOPERATIONS>
```

```
        <InitializeFromCommandline name="init"/>
        <IncludeCurrent name="keep_curr"/>
        <ReadResfile name="resfile" filename="%%resfile%%" />
        <OperateOnResidueSubset name="cavity_n_near_cavity_TO"  selector="cavity_n_near_cavity" >
           <PreventRepackingRLT/>
        </OperateOnResidueSubset>

        <OperateOnResidueSubset name="non_cavity_n_near_cavity_TO"
selector="non_cavity_n_near_cavity" >
           <PreventRepackingRLT/>
        </OperateOnResidueSubset>

        <OperateOnResidueSubset name="cavity_TO"  selector="cavity" >
           <RestrictToRepackingRLT/>
        </OperateOnResidueSubset>

        <OperateOnResidueSubset name="non_cavity_TO" selector="non_cavity" >
           <PreventRepackingRLT/>
        </OperateOnResidueSubset>

        <OperateOnResidueSubset name="FR_repack_shell_TO"  selector="2nd_shell" >
           <RestrictToRepackingRLT/>
        </OperateOnResidueSubset>

        <OperateOnResidueSubset name="non_FR_repack_shell_TO" selector="non_2nd_shell" >
           <PreventRepackingRLT/>
        </OperateOnResidueSubset>


        <OperateOnResidueSubset name="csts" selector="cavity_n_near_cavity" >
           <PreventRepackingRLT/>
         </OperateOnResidueSubset>

    </TASKOPERATIONS>

    <FILTERS>
         <TaskAwareScoreType name="cavity_score" task_operations="non_cavity_TO" scorefxn="myscore"
score_type="total_score" threshold="50000" mode="total" write2pdb="1" bb_bb="1" />

         <TaskAwareScoreType name="2nd_shell_score" task_operations="non_FR_repack_shell_TO"
scorefxn="myscore" score_type="total_score" threshold="50000" mode="total" write2pdb="1"
bb_bb="1" />
       <ScoreType name="total_score" scorefxn="myscore" score_type="total_score" threshold="50000" />
#just total_score


    </FILTERS>
```

```
    <MOVERS>

        <AtomCoordinateCstMover name="poseCST" coord_dev="0.2" bounded="false" bound_width="0.1"
sidechain="false" native="false" task_operations="csts" />
        <FastRelax name="fastrelax" repeats="5" scorefxn="myscore"
task_operations="init,keep_curr,resfile"/>

    </MOVERS>
    <APPLY_TO_POSE>
    </APPLY_TO_POSE>
    <PROTOCOLS>

       <Add mover="poseCST"/>
        <Add mover="fastrelax"/>
        <Add filter="total_score" />
        <Add filter="cavity_score" />
        <Add filter="2nd_shell_score" />
        Add filter="repack_score" />

    </PROTOCOLS>
</ROSETTASCRIPTS>
```

Section 2: Rosetta Design

Following are scripts and files used in the RosettaDesign protocol:

Input PDB file is provided as separate file:

2p16_cp_new_noligand_noligand_37_0001.pdb.gz

Command_des_noligand.sh

```
for i in {1..50}
    do
        (bash noligand_des.sh 2p16_cp_new_noligand_noligand_37_0001.pdb.gz _des_noligand_$i >>
2p16_des_noligand_Log.txt) &
    done
```

noligand_des.sh

```
RosettaPath/bin/rosetta_scripts.linuxgccrelease @noligand_FR_general.flags -s $1 -out:pdb_gz true -
suffix $2
```

noligand_des_general.flags

```
-parser:protocol path_to_XML/DesignSimpleFRFX_noligand.xml
-packing
 -ex1
 -ex2
#-ex2aro
#-ex1aro
 -extrachi_cutoff 0
 -use_input_sc
-run:preserve_header
-overwrite
-database path_to_rosetta_database/
-score:weights beta_nov16_cst
-in:ignore_unrecognized_res
-out::file::output_virtual
-ignore_zero_occupancy false
-restore_talaris_behavior
-out:nstruct 1
```

DesignSimpleFRFX_noligand.xml

```
<ROSETTASCRIPTS>
   <SCOREFXNS>

      <ScoreFunction name="myscore" weights="talaris2013_cst.wts" symmetric="0">
         <Reweight scoretype="coordinate_constraint" weight="10.0" />
      </ScoreFunction>

   </SCOREFXNS>
   <RESIDUE_SELECTORS>

      <Index name="cavity" resnums="1,2,27,80-86,127,131,132,134-137,139,146,148,160-165,168-
172,178-184,186-189,202-211,214-219" />
      <Neighborhood name="near_cavity" selector="cavity" distance="10.0" />
      <Not name="non_cavity" selector="cavity" />
      <Or name="cavity_n_near_cavity" selectors="cavity,near_cavity" />
      <Not name="non_cavity_n_near_cavity" selector="cavity_n_near_cavity" />
      <And name="2nd_shell" selectors="non_cavity,cavity_n_near_cavity" />
      <Not name="non_2nd_shell" selector="2nd_shell" />

   </RESIDUE_SELECTORS>

   <TASKOPERATIONS>

      <InitializeFromCommandline name="init"/>
      <IncludeCurrent name="keep_curr"/>
      <OperateOnResidueSubset name="cavity_n_near_cavity_TO"  selector="cavity_n_near_cavity" >
```

```
          <PreventRepackingRLT/>
       </OperateOnResidueSubset>

       <OperateOnResidueSubset name="non_cavity_n_near_cavity_TO"
selector="non_cavity_n_near_cavity" >
          <PreventRepackingRLT/>
       </OperateOnResidueSubset>

       <OperateOnResidueSubset name="cavity_TO"  selector="cavity" >
          <RestrictToRepackingRLT/>
       </OperateOnResidueSubset>

       <OperateOnResidueSubset name="non_cavity_TO" selector="non_cavity" >
          <PreventRepackingRLT/>
       </OperateOnResidueSubset>

       <OperateOnResidueSubset name="FR_repack_shell_TO"  selector="2nd_shell" >
          <RestrictToRepackingRLT/>
       </OperateOnResidueSubset>

       <OperateOnResidueSubset name="non_FR_repack_shell_TO" selector="non_2nd_shell" >
          <PreventRepackingRLT/>
       </OperateOnResidueSubset>


       <OperateOnResidueSubset name="csts" selector="cavity_n_near_cavity" >
          <PreventRepackingRLT/>
        </OperateOnResidueSubset>

    </TASKOPERATIONS>

    <FILTERS>
        <TaskAwareScoreType name="cavity_score" task_operations="non_cavity_TO" scorefxn="myscore"
score_type="total_score" threshold="50000" mode="total" write2pdb="1" bb_bb="1" />

        <TaskAwareScoreType name="2nd_shell_score" task_operations="non_FR_repack_shell_TO"
scorefxn="myscore" score_type="total_score" threshold="50000" mode="total" write2pdb="1"
bb_bb="1" />
      <ScoreType name="total_score" scorefxn="myscore" score_type="total_score" threshold="50000" />
      <DesignableResidues name="des_res"
task_operations="init,keep_curr,FR_repack_shell_TO,non_cavity_TO" designable="1" packable="0" />
      <DesignableResidues name="pack_res"
task_operations="init,keep_curr,FR_repack_shell_TO,non_cavity_TO" designable="0" packable="1" />
      <Rmsd name="RMSD_WT" superimpose="1" threshold="9999" >
      </Rmsd>

    </FILTERS>
    <MOVERS>
```

```
        <AtomCoordinateCstMover name="poseCST" coord_dev="0.2" bounded="false" bound_width="0.1"
sidechain="false" native="false" task_operations="csts" />
        <FastRelax name="fastrelax" repeats="10" scorefxn="myscore" disable_design="false"
task_operations="init,keep_curr,FR_repack_shell_TO,non_cavity_TO"/>

    </MOVERS>
    <APPLY_TO_POSE>
    </APPLY_TO_POSE>
    <PROTOCOLS>

        Add mover="poseCST"/>
        <Add mover="fastrelax"/>
        Add filter="des_res"/>
        Add filter="pack_res"/>
        <Add filter="total_score" />
        <Add filter="cavity_score" />
        <Add filter="2nd_shell_score" />
        <Add filter="RMSD_WT" />
        Add filter="repack_score" />

    </PROTOCOLS>
</ROSETTASCRIPTS>
```

Section 3: Rosetta Threading

Following are scripts and files used after RosettaDesign to make final models with incorporated mutations and apo FX structure:

Input PDB/res files are provided as separate files:
2p16_cp_new_noligand_noligand_37_0001.pdb.gz
RDR2noligand.res
HI_8noligand.res

Final model/score files are provided as separate files:
2p16_cp_new_noligand_noligand_37_0001_RDR2_noligand_884_0001.pdb
2p16_cp_new_noligand_noligand_37_0001_HI_8_noligand_630_0001.pdb

For RDR2 design:
sge_FXnoligand.sh

```
PDB_FILE=2p16_cp_new_noligand_noligand_37_0001.pdb.gz
```

```
OUT_DIR=output_"$SGE_TASK_LAST"_"$JOB_ID"
mkdir -p $OUT_DIR
OUT_FILE=$OUT_DIR/fr_RDR2noligandLog_"$SGE_TASK_ID".txt

APP="sh FRnoligand.sh"

time $APP $PDB_FILE RDR2noligand.res _RDR2_noligand_"$SGE_TASK_ID" > $OUT_FILE
```

Section 4: Dali Homology Based Design

For HI_8 design:
sge_FXnoligand_HI_8.sh

```
PDB_FILE=2p16_cp_new_noligand_noligand_37_0001.pdb.gz

OUT_DIR=output_"$SGE_TASK_LAST"_"$JOB_ID"
mkdir -p $OUT_DIR
OUT_FILE=$OUT_DIR/fr_HI_8noligandLog_"$SGE_TASK_ID".txt

APP="sh FRnoligand.sh"

time $APP $PDB_FILE HI_8noligand.res _HI_8_noligand_"$SGE_TASK_ID" > $OUT_FILE
```

FRnoligand.sh

```
APP RosettaPath/bin/rosetta_scripts.linuxgccrelease

$APP @noligand_general.flags -s $1 -out:pdb_gz true -parser:script_vars resfile=$2 -suffix $3
```

noligand_general.flags

```
-parser:protocol path_to_XML/noligand_FR_afterdes.xml
-packing
 -ex1
 -ex2
#-ex2aro
#-ex1aro
 -extrachi_cutoff 0
 -use_input_sc
-run:preserve_header
-overwrite
```

```
-database path_to_rosetta_database/
-score:weights beta_nov16_cst
-in:ignore_unrecognized_res
-out::file::output_virtual
-ignore_zero_occupancy false
-restore_talaris_behavior
-out:nstruct 1
```

noligand_FR_afterdes.xml

```
<ROSETTASCRIPTS>
  <SCOREFXNS>

    <ScoreFunction name="myscore" weights="talaris2013_cst.wts" symmetric="0">
      <Reweight scoretype="coordinate_constraint" weight="10.0" />
    </ScoreFunction>

  </SCOREFXNS>
  <RESIDUE_SELECTORS>

    <Index name="cavity" resnums="1,2,27,80-86,127,131,132,134-137,139,146,148,160-165,168-
172,178-184,186-189,202-211,214-219" />
    <Neighborhood name="near_cavity" selector="cavity" distance="10.0" />
    <Not name="non_cavity" selector="cavity" />
    <Or name="cavity_n_near_cavity" selectors="cavity,near_cavity" />
    <Not name="non_cavity_n_near_cavity" selector="cavity_n_near_cavity" />
    <And name="2nd_shell" selectors="non_cavity,cavity_n_near_cavity" />
    <Not name="non_2nd_shell" selector="2nd_shell" />

  </RESIDUE_SELECTORS>

  <TASKOPERATIONS>

    <InitializeFromCommandline name="init"/>
    <IncludeCurrent name="keep_curr"/>
    <ReadResfile name="resfile" filename="%%resfile%%" />
    <OperateOnResidueSubset name="cavity_n_near_cavity_TO"  selector="cavity_n_near_cavity" >
      <PreventRepackingRLT/>
    </OperateOnResidueSubset>

    <OperateOnResidueSubset name="non_cavity_n_near_cavity_TO"
selector="non_cavity_n_near_cavity" >
      <PreventRepackingRLT/>
    </OperateOnResidueSubset>

    <OperateOnResidueSubset name="cavity_TO"  selector="cavity" >
      <RestrictToRepackingRLT/>
```

```
        </OperateOnResidueSubset>

        <OperateOnResidueSubset name="non_cavity_TO" selector="non_cavity" >
           <PreventRepackingRLT/>
        </OperateOnResidueSubset>

        <OperateOnResidueSubset name="FR_repack_shell_TO"  selector="2nd_shell" >
           <RestrictToRepackingRLT/>
        </OperateOnResidueSubset>

        <OperateOnResidueSubset name="non_FR_repack_shell_TO" selector="non_2nd_shell" >
           <PreventRepackingRLT/>
        </OperateOnResidueSubset>


        <OperateOnResidueSubset name="csts" selector="cavity_n_near_cavity" >
           <PreventRepackingRLT/>
         </OperateOnResidueSubset>

    </TASKOPERATIONS>

    <FILTERS>
         <TaskAwareScoreType name="cavity_score" task_operations="non_cavity_TO" scorefxn="myscore"
score_type="total_score" threshold="50000" mode="total" write2pdb="1" bb_bb="1" />

         <TaskAwareScoreType name="2nd_shell_score" task_operations="non_FR_repack_shell_TO"
scorefxn="myscore" score_type="total_score" threshold="50000" mode="total" write2pdb="1"
bb_bb="1" />
       <ScoreType name="total_score" scorefxn="myscore" score_type="total_score" threshold="50000" />
       <DesignableResidues name="des_res"
task_operations="init,keep_curr,FR_repack_shell_TO,non_cavity_TO" designable="1" packable="0" />
       <DesignableResidues name="pack_res"
task_operations="init,keep_curr,FR_repack_shell_TO,non_cavity_TO" designable="0" packable="1" />
       <Rmsd name="RMSD_WT" superimpose="1" threshold="9999" >
       </Rmsd>

    </FILTERS>

<MOVERS>

        <AtomCoordinateCstMover name="poseCST" coord_dev="0.2" bounded="false" bound_width="0.1"
sidechain="false" native="false" task_operations="csts" />
        <FastRelax name="fastrelax" repeats="10" scorefxn="myscore" disable_design="false"
task_operations="init,keep_curr,resfile"/>

    </MOVERS>
    <APPLY_TO_POSE>
    </APPLY_TO_POSE>
```

```
  <PROTOCOLS>

      Add mover="poseCST"/>
      <Add mover="fastrelax"/>
      Add filter="des_res"/>
      Add filter="pack_res"/>
      <Add filter="total_score" />
      <Add filter="cavity_score" />
      <Add filter="2nd_shell_score" />
      <Add filter="RMSD_WT" />

  </PROTOCOLS>
</ROSETTASCRIPTS>
```

RDR2noligand.res

```
USE_INPUT_SC
EX 1 EX 2
start
  1  A  NATAA
  2  A  NATAA
.
.
205  A  PIKAA A
.
.
```

HI_8noligand.res

```
USE_INPUT_SC
EX 1 EX 2
start
  1  A  NATAA
  2  A  NATAA
.
.
 78  A  PIKAA S
 79  A  PIKAA T
 80  A  PIKAA Y
 81  A  PIKAA V
 82  A  PIKAA P
 83  A  PIKAA G
```
